# Delivery of the second twin: influence of presentation on neonatal outcome, a case controlled study

**DOI:** 10.1186/s12884-018-1815-0

**Published:** 2018-05-18

**Authors:** Gerhard Bogner, Valentina Wallner, Claudius Fazelnia, Martina Strobl, Birgit Volgger, Thorsten Fischer, Volker R. Jacobs

**Affiliations:** 1 Department of Obstetrics and Gynecology (OB/GYN), Paracelsus Medical University, Muellner Hauptstr. 48, A-5020 Salzburg, Austria; 2 Paracelsus Medical University, Salzburg, Austria; 3 Department of Obstetrics and Gynecology, Hospital of Wels-Grieskirchen, Wels, Austria; 4 Department of Obstetrics and Gynecology, Hospital Lienz, Lienz, Tyrol Austria

**Keywords:** Breech delivery, Delivery of second twin, Management of twin delivery, Outcome second twin, Mode of delivery, Non-cephalic second twin

## Abstract

**Background:**

Spontaneous vaginal twin delivery after 32nd week of gestation is safe when first twin presenting cephalic. Aim of this study is to identify obstetric factors influencing the condition of second twin and to verify whether non-cephalic presentation and vaginal breech delivery of the second twin is safe.

**Methods:**

This is a retrospective case controlled cohort study of 717 uncomplicated twin deliveries ≥32 + 0 weeks of gestation from 2005 to 2014 in two tertiary perinatal centers. Obstetric parameters were evaluated in three groups with descriptive, univariate logistic regression analysis for perinatal outcome of second twins.

**Results:**

The three groups included twins delivered by elective cesarean section ECS (*n* = 277, 38.6%), by unplanned cesarean section UPC (*n* = 233, 32.5%) and vaginally (*n* = 207, 28.9%). Serious adverse fetal outcome is rare and we found no differences between the groups. Second twins after ECS had significant better umbilical artery UA pH (*p* < 0.001) and better Apgar compared to UPC (*p* = 0.002). Variables for a fetal population “at risk” for adverse neonatal outcome after vaginal delivery (UA pH < 7.20, Apgar 5´ < 9) were associated with higher gestational age (*p* = 0.001), longer twin-twin interval (*p* = 0.05) and vacuum extraction of twin A (*p* = 0.04). Non-cephalic presentation of second twins was not associated (UA pH < 7.20 OR 1.97, CI 95% 0.93–4.22, *p* = 0.07, Apgar 5´ < 9 OR 1.63, CI 95% 0.70–3.77, *p* = 0.25, Transfer to neonatal intermediate care unit *p* = 0.48). Twenty-one second twins (2,9%) were delivered by cesarean section following vaginal delivery of the first twin. Even though non-cephalic presentation was overrepresented in this subgroup, outcome variables were not significantly different compared to cephalic presentation.

**Conclusions:**

Even though elective cesarean means reduced stress for second twins this seems not to be clinically relevant. Non-cephalic presentation of the second twin does not significantly influence the perinatal outcome of the second twin but might be a risk factor for vaginal-cesarean birth.

## Key message

Second twins after vaginal breech delivery are adapted as well as cephalics. Non-cephalic presentation of the second twin is a risk factor for combined vaginal-cesarean birth.

## Background

Twin pregnancies are rising during the past decades in Europe and the U.S. [1, 2] Currently 2–3% of all births are twin births [3]. At the same time the rate of cesarean section in twin pregnancies has increased globally [4, 5]. A prospective randomized trial showed that vaginal delivery in uncomplicated twin pregnancies after 32 + 0 gestational weeks is possible and safe if the first twin is in cephalic position [6]. Thus, in these cases it is recommended that women be counseled to attempt vaginal delivery [7].

Nevertheless, the delivery of the second twin remains a challenge, especially if the twin B is not in cephalic presentation. Generally, retrospective studies show increased risk of perinatal mortality for the second twin [8, 9]. Only one small randomized trial suggests that in twins with non-cephalic presentation after the thirty-fifth gestational week the neonatal outcome of the second twin is not significantly influenced by the route of delivery [10]. Due to lacking prospective studies to choose the best mode of delivery on the basis of individual case characteristics, in one hand expertise in the management of vaginal twin delivery is mandatory and on the other hand patient preference has to be respected [11]. A trial of labor is successful in 77% [12].

Due to a shift towards Caesarean Section for singleton breech, particularly since the publication of the Term Breech Trial, experience in managing vaginal breech deliveries in Europe has markedly decreased [13–16]. For vaginal breech deliveries a variety of standard maneuvers are described in medical textbooks [17]. However, there is still no evidence-based data for these techniques available until now. Active second stage management for the second twin is considered to be associated with better neonatal outcome and a low rate of combined vaginal-cesarean delivery [18]. In recent years some working groups have clinically implemented all fours position of the mother for vaginal breech delivery in singletons [19]. Up to date, experiences with vaginal breech delivery of the second twin in all fours have not been published.

Aim of this study was to describe long-term experience of two tertiary perinatal centers with twin delivery, generally. Primary objective is to show whether attempted vaginal delivery of twins without additional risk is associated with serious fetal adverse events for the second twin. Furthermore, the purpose was to identify obstetric variables as potential risks for asphyxia of the second twin. Special focus was set on breech presentation of the second twin. Secondary aim was to present experience with delivery management and outcome of second breech twins in all-fours position of the mother.

## Methods

This is a retrospective analysis of 717 twin births from January 1st 2005 to October 31st 2014 in two European tertiary obstetric perinatal centers. Data were extracted from the medical records and the obstetric clinical database. Only twin births without any additional maternal or fetal risks were included. Inclusion criteria were completed 32nd week of gestation, cephalic presentation of the first twin (for vaginal attempt). Exclusion criteria were perinatal complications such as HELLP syndrome, preeclampsia, growth restrictions <5th percentile, known fetal malformations and pre-labor stillbirth of one twin.

According to modes of delivery three main groups were stratified as

1. Elective (planned) Cesarean section [ECS],
2. Unplanned Cesarean section [UPC] and
3. Vaginal delivery [VD].

**Fig. 1:**
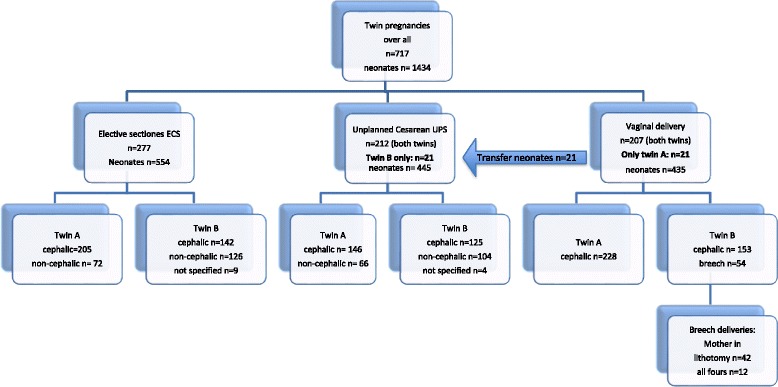
Design of the study: Three groups of mode of twin delivery to compare outcome of twins B. Out of 207 twin deliveries intended to deliver vaginally *n* = 21 twin B were born by C-section after vaginal delivery of twin A. Their outcome measures were analyzed in the UPC group

ECS were intended cesarean without onset of labor and/or rupture of membranes. UPC summarized all deliveries ended with abdominal delivery of at least one child. Subgroups were cephalic and non-cephalic presentation (Fig. 1). The perinatal outcome of second twins was calculated statistically between these three groups. Subgroup analysis focused on vaginal delivery (*n* = 207) and non-cephalic presentation of the second twin (*n* = 54).

Primary outcome variable was serious adverse fetal events defined by birth pH of the umbilical artery UA pH < 7.10 and Apgar after 5 min < 6 and transfer to neonatal intensive care unit NICU due to fetal distress, severe fetal injury or death.

Secondary outcome variable as surrogate for fetus “at risk” for asphyxia was defined as UA pH < 7.20, Apgar after 5 min < 9 or postpartal transfer of the newborn to NICU due to perinatal fetal distress.

Special focus of this study was set on all cases of twin deliveries with second twins in breech presentation combined with attempt of vaginal delivery on all fours position of the mother. The same obstetrician attended all these deliveries. They were documented prospectively.

### Breech delivery procedure (including on all fours)

Vaginal delivery of second twin is managed and intended not active. The process of vaginal delivery of the second twin with non-cephalic presentation is summarized in the following: After delivery of the first twin, concomitant oxytocin infusion support is stopped. Then the second twin’s presentation is checked by ultrasound in maternal supine position. In case of unstable or incomplete breech presentation, the obstetrician is using his flat hands for stabilization of fetal position laterally under continuous fetal heart rate monitoring. At this point the obstetrician is waiting for contractions. In case of transverse presentation, the fetus is turned into breech or cephalic presentation by an external version and stabilized manually under ultrasound guidance. The technique is according to external cephalic version and was previously described [20]. After fetal position is stabilized, an amniotomy is performed at re-starting of spontaneous contractions. The target of the timeline is to deliver both children within an interval of less than 30 min. Vigilant monitoring for abnormal fetal heart rate or vaginal bleeding as an indicator for placental abruption is required. Ongoing watchful waiting is mandatory in case of a prolonged time period of > 30 min between the two twins [21]. After rupture of membranes a vaginal examination is required to exclude an umbilical cord prolapse. Subsequently, the obstetrician waits for the fetal bottom to extend and rotate under continuous fetal heart rate monitoring. Optionally, oxytocin infusion is given to support manual maneuvers to delivery the fetus. Choice of maneuvers was at sole discretion of the obstetrician.

If all-fours management is intended, no later than this point the women is instructed to change into all-fours position. Without any interference by obstetrician and midwife are watching for the spontaneous process of delivery. No episiotomy, no manual perineal support or fetal extraction is intended. Labor and gravity are the only factors actively progressing the delivery. When delivery of shoulder and arms is delayed, vaginal delivery is completed in supine position using the standard maneuvers to deliver neonates (partial or complete breech extraction, Mauriceau maneuver, Bracht maneuver, movement of nuchal arms, etc.). In case of delay in spontaneous delivery of the head in all fours, a bolus of 3 IU/ml oxytocin is applied. If the fetal head does not appear within up to three contractions, fetal head is delivered over the perineum by bilateral thumb pressure on the shoulders of the fetus towards the maternal symphysis. Technique and process of delivery in all fours has recently been described [19]. Instant standby of anesthesia and neonatologist is provided for potential emergency C-section of the second twin during entire vaginal delivery attempt.

### EC approval

Although twin birth and vaginal breech delivery is a commonly accepted mode of delivery and standard of care in singletons and twins, the study protocol was reviewed and approved by the local Ethics Committee (415-EP/73/145–2012, Ethics Committee for the State of Salzburg, Austria).

#### Statistical Methods

The data were summarized using standard methods of descriptive statistics. Means of metric data were compared by t-tests (2 groups) or ANOVA (3 groups). Categorical data was compared with chi-squared tests when enough data was available; Fisher’s exact test was used for sparse data. Analysis of the relationship between binary outcome variables with various risk factors was carried out by logistic regression. All calculations were performed using statistical software package SPSS version 18 (SPSS Inc. Chicago, IL, USA). The significance level was set to 5% (α = 0.05).

## Results

Of all twin births (*n* = 717, *n* = 1434 children) *n* = 277 s twins (38.6%) were delivered by ECS and *n* = 233 (32.5%) by UPC due to different reasons such as early onset of labor, rupture of membranes and trial of labor, including 21 neonates (2.9%) born by cesarean section after vaginal delivery of twin A. Main reasons for UPC were preterm delivery < 35 week of gestation (*n* = 69, 25.8%), intended ECS and early onset of labor (*n* = 115, 49.3%) and termination after trail of labor because of obstetrical problems (*n* = 37, 15.8%). In the third group 207 women (28.9%) delivered vaginally (VD). In this group *n* = 153 twin B (73.9%) presented in cephalic and *n* = 54 (26.1%) in breech, finally. The three groups differ significantly in gestational age (35.8 ± 1.6; 35.0 ± 1.9; 36.2 ± 1.7 weeks of gestation, *p* < 0.001) and parity (1.6 ± 0.9; 1.5 ± 0.9; 2.0 ± 1.0; difference between C-section and VD *p* < 0.001, no difference between the two C-section groups *p* = 0.82) (Table 1).

**Table 1:** Comparison by three groups of mode of delivery groups for the second twin: elective cesarean section ECS, unplanned cesarean section UPC and vaginal delivery VD

	planned C-section (ECS) *n* = 277	Unplanned C-Section (UPC) *n* = 233	Vaginal delivery (VD) *n* = 207	*P*-values (ECS vs. UPC, ECS vs. VD, UPC vs. VD)
Age of the mother (years)	32 ± 5.0	31.5 ± 5.7	31.4 ± 4.9	ns. (*p* = 0.43)
Gestational age (weeks)	35.8 ± 1.6	35.0 ± 1.9	36.2 ± 1.7	< 0.001, 0.012, < 0.001
Parity n	1.6 ± 0.9	1.5 ± 0.9	2.0 ± 1.0	0.82, < 0.001, < 0.001
Gender m/f (%)	118/159 (42.6/53.4)	110/123 (47.2/52.8)	100/107 (48.3/51.7)	ns.
Chorionicity M/D (%)	64/211 (23.1/76.9)	49/183 (21.0/79.0)	44/137 (21.2/78.8)	ns.
Previous C-section	18 (6,5%)	9 (3.9%)	0	
Presentation:				
Vertex	142 (51.3%)	125 (53.6%)	153 (73.9%)	< 0.001
Breech	75 (27.1%)	59 (25.3%)	54 (26.1%)	
Transverse	51 (18.4%)	45 (19.3%)		
Not applicable	9	4		
Lung maturation	55 (19.9%)	35 (15.0%)	16 (7.7%)	0.001
Induction of labor	4 (1.4%)	40 (17.2%)	99 (47.8%)	< 0.001

Continuous data are given by mean and+/− standard deviation

Serious adverse outcome of the second twin was rare. There was no severe fetal injury, no fetal death within the first 4 weeks after delivery. Postpartally three minor fetal malformations were diagnosed. Only two second twin after UPC and one after vaginal delivery showed decreased Apgar 5` < 6, UA pH < 7.10 and transfer to NICU (asphyxia). Three first twins showed asphyxia (two with UPC, one twin after vacuum delivery). There were seven newborns with UA pH < 7.0 (three twin A, four twin B – two of them after UPC, one after vacuum extraction, one by emergency C-section of the second twin).

Paired comparison of the first and the second twin shows that UA pH is reduced in second twins (*p* = 0.003). There was no difference between the twins regarding Apgar and transfer rate to NICU (*p* = 0.36 and *p* = 0.26, respectively). If vaginal delivery of the first twin is followed by surgical delivery of the second twin, all outcome parameter of the second twin were decreased compared to parameters for the first twin (for all *p* < 0.001).

Focused comparison of the variables of neonatal outcome of second twin showed significantly lower Apgar at 5′ < 9 (*p* = 0.003) following UPC, number of neonates with UA pH < 7.20 was significantly higher following UPC and VD (*p* < 0.001). Postpartal Apgar at 5′ > 9 and the transfer rate to the NICU were increased after UPC (*p* < 0.001), generally. Compared to VD (*n* = 6/207) UPC showed an increased number of transfers due to neonatal distress or respiratory problems (*n* = 17/233; *p* = 0.04) as well (Table 2). Postpartal UA pH was significantly lower in VD group (7.26 ± 0.08, *p* < 0.001) but without a higher transfer rate of newborns to NICU due to clinical respiratory distress (*p* = 0.07). Within the VD group Table 3 shows the comparison of variables in the subgroups cephalic (*n* = 153) and breech presentation (*n* = 54). Significant differences are seen in gestational age (*p* = 0.02), twin-twin interval (*p* = 0.04), Apgar 5′(*p* = 0.05) and UA pH values (*p* = 0.30).

**Table 2:** Fetal outcome of second twin according to mode of delivery

		Elective C-Section (ESC) *n* = 277	Unplanned C-Section (UPC) *n* = 233	Vaginal delivery (VD) *n* = 207	*P*-value Anova
Gestational week	32–35	93 (33.6%)	140 (60.1%)	65 (31.4%)	< 0.001
	36–39	184 (66.4%)	93 (39.9%)	142 (68.6%)	
Fetal weight [g]		2351 ± 450	2207 ± 414	2518 ± 400	< 0.001
Head circumference [cm]		32.6 ± 1.6	32.2 ± 1.6	33.0 ± 1.3	< 0.001
Apgar 5′ mean		9.47 ± 0.8	9.22 ± 1.0	9.44 ± 0.9	0.005
Apgar 5′ (n)	< 6	1	0	2	0.003
	6–8	26	48	28	
UA pH	mean	7.31 ± 0.05	7.29 ± 0.08	7.26 ± 0.08	< 0.001
UA pH	< 7.10	0	6	7	< 0.001
	< 7.20	4	16	30	
Transfer to NICU *n* = 219	all	72 (26.0%)	109 (46.8%)	38 (18.4%)	< 0.001
due to fetal distress		11 (4.0%)	17 (7.3%)	6 (2.9%)	Anova *p* = 0.07 0.04 UPC vs. VD

Continuous data are given by mean and+/− standard deviation

**Table 3:** Within the VD group, comparison of variables of the subgroups cephalic (*n* = 153) and non-cephalic (*n* = 54) presentation

	Cephalic *n* = 153	Breech *n* = 54	*p*-value
Age of the mother (years)	31.2 ± 5.2	32.0 ± 3.9	ns (0.22)
Parity n			
1	67 (43,8%)	10 (18,5%)	0.002
2+	76 (56,2%)	44(81,5%)	
Gestational age (weeks)	36.1 ± 1.4	36.7 ± 1.2	0.007
32–35	56 (36,6%)	9 (16,7%)	
36–40	97 (63,4%)	45 (83,3%)	
Gender m/f (%)	65/88 (42.5/53.5)	35/19 (64.8/35.2)	*p* = 0.005
Chorionicity (%) mono/di/na	26/103/24 (17.0/67.3/15.7)	18/34/2 (33.3/62.0/3.7)	*p* = 0.04
Lung maturition n	9	7	ns (*p* = 0.09)
Induction of labor n	70 (45,7%)	29 (53,7%)	ns (*p* = 0.32)
Twin-twin interval (min)	13.2 ± 10.8	17.02 ± 12.7	0.04
Birth weight (g)	2494 ± 392	2577 ± 409	0.19
Head cirumference (cm)	32.9 ± 1.4	33.1 ± 1.1	0.48
Apgar 5 (n)			
6–8	18	8	ns (*p* = 0.2)
< 6	1	1	
UA pH	7.27 ± 0.08	7.24 ± 0.08	0.03
< 7.10 (n)	7	1	
7.10–7.20 (n)	18	16	
Transfer to NICU (n) due to distress	30	8	ns (*p* = 0.5)
	4	2	

Continuous data are given by mean and+/− standard deviation

Univariate analysis of parameter of second twins (*n* = 207) in the VD group revealed a significant association of “at risk” outcome variables: Postpartal UA pH < 7.20 was significantly associated to gestational age (OR = 1.98 CI 95% 1.19–2.05, *p* = 0.001), longer twin-twin interval (OR 1.03, CI 95% 1.0–1.06, *p* = 0.05) and vacuum extraction of the first twin (OR 2.7, CI 95% 1.05–6.96, *p* = 0.04) (Table 4). In univariate analysis for Apgar 5´ < 9 only two explanatory variably display significant effect on the outcome variable: Gestational age (OR = 0.63, CI 95% 0.5–0.8, *p* < 0.001) and derived birth weight (OR = 0.87, CI 95% 0.79–0.97). The association was higher after the 36th week of gestation than between the 32nd and 35th week of gestation (UA pH *p* = 0.003, Apgar 5´ < 9 *p* = 0.08). In particular, there was no association between presentation of the second twin and neonatal outcome (UA pH < 7.20 OR 1.97, CI 95% 0.93–4.22, *p* = 0.07, Apgar 5´ < 9 OR 1.63, CI 95% 0,70–3.768, *p* = 0.25).

**Table 4:**
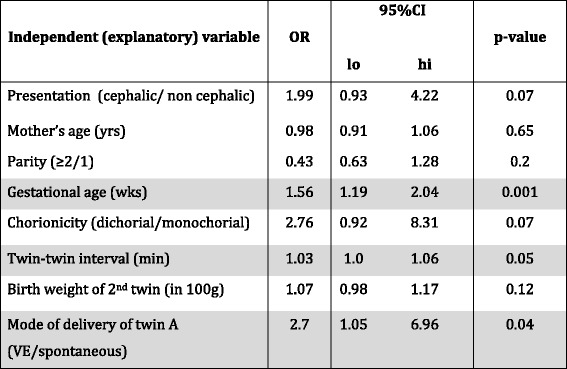
Vaginal delivery of the second twin (*n* = 207) and outcome evaluation by univariate analysis logistic regression of dependent (neonatal variables umbilical artery pH < 7.20) and independent (obstetrical) factors

Correlation and statistically significant differences are highlighted

Among *n* = 440 intended or initiated vaginal deliveries *n* = 21 neonates (4.8%) delivered after UPC following VD of the first twin. Of these, *n* = 11 were in non-cephalic presentation (five breeches, six transverse presentation). The outcome parameters of these children were significantly lower compared to other neonates after UPC (UA pH *p* < 0.001, Apgar 5′ *p* < 0.001, neonatal transfer *p* = 0.03). In this situation there was a weak correlation between presentation of the 2nd twins in non-cephalic presentation and this “at risk” outcome parameter (UA pH *p* = 0.054, Apgar 5′ *p* = 0.97, neonatal transfer *p* = 0.3).

### Vaginal breech delivery on all fours

Within the VD group (*n* = 207) *n* = 54 were born in breech position. From these *n* = 12 women (22.2%) attempted breech delivery in maternal all-fours position. Six of these deliveries (50%) were completed spontaneously without any intervention of the obstetrician. In three additional interventions were successful and birth was completed in all fours. Only three women were converted back to supine position and delivered by standard maneuvers (two due to a failure of progress and/or abnormal fetal heart rate and one due to maternal indication because of shoulder complaints).

## Discussion

The results of this study suggest that presentation of the second twin does not significantly influences its clinical obstetrical outcome. It has been shown that non-cephalic second twins are overrepresented in twin deliveries with combined vaginal/cesarean procedure. Furthermore, there was no significant correlation between presentation and adverse perinatal outcome after UPC of the second twin.

Within the VD group there were significant differences in the subgroups of cephalic and breech deliveries regarding twin-twin interval, Apgar 5’values and UA pH values. This fact is due to our non-active management of the second twin and seems to be not clinically relevant because there are no differences in the number of severe poor values of Apgar 5′ < 6 and UA pH < 7.10 in the subgroup of neonates after breech delivery.

The subgroup analysis of vaginal breech delivery attempted in all fours seems to be feasible and was completed in 9 of 12 attempts. In the three attempts obstetric skills and training in management of twins and breeches were essential to complete the vaginal delivery. To our knowledge this is the first study presenting a case series of vaginal breech delivery of the second twin in the all fours position of the mother. We were aware that delivery of the second twin in maternal all-fours position is contradictory to active management. Therefore, the methode has the potential to prolong the inter- twin delivery interval, to increase risk for difficult obstetric maneuvers and and thus cesarean delivery for the second twin. Even we had no problems with this 12 all fours, the small sample size does not allow any conclusions to be drawn about safety or likelihood of vaginal birth in comparison to lithotomy position.

It is obvious for the authors that pH < 7.20 and Apgar 5′ < 9 is not regarded as adverse neonatal outcome. But there were only few neonates with general used definitions of severe perinatal asphyxia UA pH < 7.10 and Apgar 5′ < 6 in our study. Therefore setting cut off values higher includes neonates who may be at risk or tend to neonatal asphyxia and was used as a secondary outcome. To show differences in serious adverse outcome sample volume of twin deliveries are too small.

UPC summarized all started deliveries ended with abdominal delivery of at least one child. Due to the retrospective design of the study it is not possible to distinguish clearly between UPC with starting contracting, premature rupture of membranes either, and failed trail of labor. At one side women presenting first time (i.e. preterm) at the department with started labor and no documented intention for mode of delivery. On the other side there are women who intended vaginal delivery and changed their decision to C-Section. Women with obvious intention for ECS and prematurely slightly starting without cervix dilatation were put in the ECS group.

The Twin Birth Study, a prospective multicenter randomized study, published in 2013, with 105 participating hospitals in 25 countries, showed no difference between neonatal and maternal mortality and morbidity under optimal conditions when delivery was planned vaginally or as cesarean section [6]. Detailed prospective analyses of data that would help in decision making for best mode of delivery and obstetric management in vaginal delivery of second twins have not been published yet. Therefore, only retrospective studies serve as guidance [11].

Recently a retrospective study with large sample size pointed out that planned cesarean section lowers the risk of serious neonatal morbidity, in particular of the second twin. Notably it favors planned cesarean at gestational week ≥36 [22]. Accordingly, our data suggests that higher gestational age may be a risk factor for neonatal distress of the second twin.

Non-cephalic presentation of the second twin at term is infrequent, with about approximately 25% of all twin pregnancies, and planned cesarean section is more common in the non-cephalic presentation [23]. Intrapartum change from cephalic to non-cephalic during delivery was stated in 11% [24]. Due to declining expertise in perinatal management of vaginal breech deliveries, a safe method to deliver the second twin is desired. The data from this study support that vaginal breech delivery in all fours is feasible and an option for management of the second twins [19].

For reasons of better management in delivering the second twin supine position has been established as superior maternal position for the obstetrician [25]. Hypothetically, the upright position of the mother (sitting or in all fours) is a more natural and physiological delivery position, but the obstetrician has limited possibility to intervene. Breech deliveries in all fours might be an option for skilled obstetricians, but is not adequate for settings with little or no experience and without continuous clinical practicing facilities.

For evidence-based counseling of women for vaginal birth in twin pregnancies, there are some risks to consider adequately such as presentation, chorionicity, birth interval to twin A or weight differences. Non–cephalic presentation is not considered a risk factor for failed trial of labor, but only as a risk factor for a combined mode of delivery (vaginal-cesarean). In literature risk factors that were associated independently with cesarean delivery were primiparity (OR 5.78; 95% CI 2.24–14.88) and advanced maternal age of ≥35 years (OR 2.36; 95% CI, 1.16–4.80). Trial of labor was successful in 77–82% [12]. Even women at highest risk for cesarean delivery (nulliparous, advanced maternal age, induced labor) still had a probability of 48.6% for vaginal delivery [26]. Our data show that maternal age and low parity is an elective factor for ECS and probably biased the selection for this study. We could not confirm that age of the mother and parity is a risk factor for adverse neonatal outcome.

Regarding studies focused on non-cephalic presentation of the second twin, there is only one prospective randomized trial not reporting any significant differences in neonatal morbidity [10]. A recently published retrospective secondary data set analysis of the WHO Global Survey on Maternal and Perinatal Health focused on non-cephalic presentation of the second twin and revealed only an association with increased odds of Apgar < 7 at 5 min, but not of any other maternal/perinatal outcomes [23]. They conclude - together with others [27] - that a non-cephalic presentation is not relevant when considering planning the mode of delivery.

Data from this study indicates that combined vaginal-cesarean birth is the least optimal method for 2nd twins and should be avoided if possible [25]. Vaginal-cesarean delivery is associated with non-cephalic second twin and a prolonged inter-delivery interval [28]. There is evidence that active management of second stage of labor, including breech extraction of second twins and internal version of non-engaged second twins, is resulting in avoidance of cesarean delivery for the second twin after vaginal delivery of the first twin [18, 29]. Without active management of the second stage, the likelihood of a combined vaginal-cesarean delivery can be as high as 6–10% [30]. In contrast, delivery (cephalic and non cephalic) in this setting and especially in all fours is intended as non-interventional spontaneous and therefore it is not in accordance with active management.

Additionally, monochorionic twins are considered to have an increased risk for fetal death (even at term), necrotizing enterocolitis and neuromorbidity [31]. A recent retrospective study did not indicate monochorionicity as an additional risk for vaginal birth [32]. A delivery prior to the end of gestational week 36 is not justified in uncomplicated monochorionic pregnancies [33]. Therefore, the optimal time of delivery in monochorionic twins is the completed 37th week of gestation, in dichorionic twins the completed 38th week of gestation [34]. Our data approved monochorionicity without evidence of feto-fetal transfusion syndrome is not an indication for elective cesarean section. Even in monochorionic twin deliveries, a trial of labor succeeds in 77% without increased perinatal morbidity [31, 33].

Regarding the influence of birth interval between twin A and B, breech presentation of twin B has the potential to decrease the perinatal outcome (higher rate of vaginal-cesarean delivery but without decrease of post partum UA pH [9, 18, 35]).

Strengths of this study are the large sample size of twin deliveries in tertiary perinatal centers with equal group size of mode of delivery and narrow evidence-based inclusion criteria. The study also includes a high number of twin births with homogeneous processes of obstetrical management. Innovative is the subanalysis of breech deliveries in all fours as a potential new option of management in twin delivery. Limitations are the retrospective study design and non-randomization as well as the rather small number of women with vaginal breech delivery due to the rarity of breech presentation in second twins.

## Conclusions

Despite elective Cesarean in twin pregnancies without maternal and neonatal risk factors shows better primary outcome this seems clinically not relevant when twin A is presented in cephalic. Even vaginal delivery of the second twin in breech presentation seems to be safe for management of the second twin after completed 32nd weeks of gestation. Non-cephalic presentation of the second twin is associated with a higher risk for combined vaginal-cesarean delivery. However, non-cephalic second twins are not exposed and are not at risk for asphyxia compared to second twins with cephalic presentation. Presentation of the second twin is not an eligible variable for counseling women for mode of delivery. Management of vaginal breech delivery for second twins in all four position of the mother seems to be feasible and an option for breech management. However, this technique might only be conducted in centers where obstetricians are experienced in breech delivery including routine training of vaginal breech delivery using an obstetric model.

## Abbreviations

95%CI: Confidence interval 95% percentile
Apgar 5′ < 9: Apgar after 5 min below value 9
ECS: planned (elective) cesarean section
NICU: Neonatal intermediate care unit
NS: Not significant
OR: Odds ratio
UA pH: pH of umbilical artery
UPC: Unplanned cesarean section
VD: Vaginal delivery
VE: Vacuum extraction
